# Meta-analysis study of the therapeutic impact of Mesenchymal stem cells derived exosomes for chronic kidney diseases

**DOI:** 10.1016/j.bbrep.2025.102072

**Published:** 2025-06-03

**Authors:** Himanshu, Gunjan, Ramendera Pati Pandey, Riya Mukherjee, Chung-Ming Chang

**Affiliations:** a Graduate Institute of Biomedical Sciences, Chang Gung University, No. 259, Wenhua 1st Road, Guishan District Taoyuan City, 33302, Taiwan, ROC; b Master & Ph.D. Program in Biotechnology Industry, Chang Gung University, No. 259, Wenhua 1st Road, Guishan District, Taoyuan City, 33302, Taiwan, ROC; c School of Health Sciences and Technology (SoHST) UPES, Bidholi, Dehradun, 248007, Uttarakhand, India; d Department of Biotechnology and Microbiology, SRM University Delhi-NCR, Sonepat, Haryana, India; e Department of Medical Biotechnology and Laboratory Science, Chang Gung University, No. 259, Wenhua 1st Road, Guishan District, Taoyuan City, 33302, Taiwan, ROC

**Keywords:** Mesenchymal stem cells, Exosome, Chronic kidney diseases, Renal fibrosis, Meta-analysis

## Abstract

Mesenchymal stem cell-derived exosomes (EXOs) represent a promising avenue for treating chronic kidney diseases (CKD), though their precise impact remains somewhat elusive. To address this gap, we conducted a systematic analysis, scouring databases and clinical trial repositories for relevant studies from 2019 to 2023. Seventeen papers were meticulously selected for their focus on mesenchymal stem cell-derived exosomes (MSC-EXOs) and their potential in CKD treatment. Our comprehensive meta-analysis, incorporating 15 preclinical and 6 clinical studies, underscores the efficacy of MSC-EXOs in improving renal function while attenuating tubular injury, inflammation, apoptosis, collagen deposition, and renal fibrosis. Notably, post-treatment with MSC-EXOs exhibited significant associations with various CKD markers, with pooled proportions indicating a considerable impact on blood urea nitrogen (BUN) and serum creatinine (SCR) levels. Subgroup analyses based on animal models further elucidated heterogeneity within the studies. In conclusion, MSC-EXOs demonstrate promise in enhancing renal function and reducing CKD risk, as evidenced by both preclinical and clinical data. Their efficacy in lowering SCR and BUN levels while enhancing filtration rate suggests MSC-EXOs as a viable and secure alternative to cell-based therapies, thereby providing valuable insights for personalized CKD treatments despite inherent limitations.

## Graphical Highlights

• Meta-analysis shows MSC-EXOs improve renal function and lower CKD risk, highlighting their strong therapeutic potential.
• Pooled data show MSC-EXO therapy significantly lowers BUN/SCR and raises GFR, supporting its therapeutic potential.
• Exosomes show a significant effect on renal function, as indicated by the study findings.

## Graphical abstract

**Figure:**
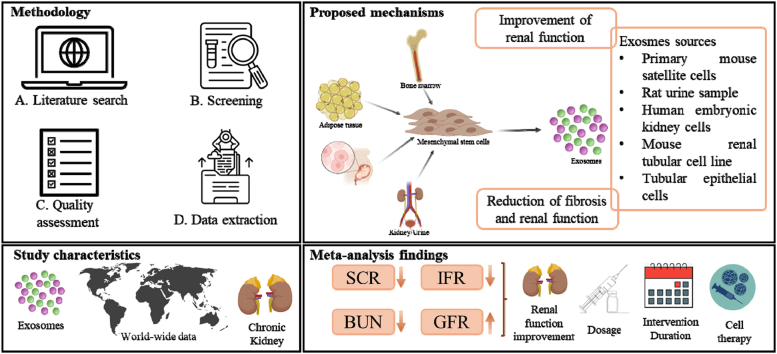

## 1 Introduction

Chronic kidney disease (CKD) emerges as a significant global health concern that demands attention. It is elevated incidence, substantial healthcare burden, insidious onset, poor prognosis, and other associated challenges pose serious threats to the well-being of individuals worldwide [1,2]. CKD emerges because of diminished oxygen delivery to the kidneys. Subsequent amplification of kidney hypoxia leads to compromised regenerative capacity, cell damage, oxidative stress, eliciting inflammatory responses and renal fibrosis within the kidney compartments [3]. To counteract these processes, numerous pharmaceutical therapies have been developed. There are five phases of CKD, and each one is linked to increased risks of cardiovascular morbidity, early mortality, and/or a poor standard of life [4]. Renal damage, indicative of CKD, encompasses pathological abnormalities, abnormal urine sediment, or an elevated urinary albumin excretion rate, detectable through imaging or renal biopsy [5]. Currently, none of these therapies have been clinically validated to effectively alter the outcome of CKD [[6], [7], [8]]. By the year 2040, it is projected to become the fifth most prevalent cause of death globally [9]. Unfortunately, conventional pharmacological methods often overlook the intricate interconnections and complexities of overlapping disease-related mechanisms [10]. An alternate approach including targeted delivery and control of disease pathways through the transfer facilitated by extracellular vesicles (EVs) could address the bottleneck between regenerative medicine and current pharmaceutical treatments [11].

According to recent data, CKD is a global health issue, affecting approximately 9 %–13 % of the worldwide population (approximately 700 million to one billion people), with a significant portion of patients falling into stage 3 of the disease [4,5,12]. It rates up to 15 % in the USA, that is about 37 million people, causing a high economic burden [12]. Tragically, millions of individuals die each year due to the lack of affordable treatment options. The 2023 ISN-GKHA report highlights the widespread impact of CKD, with around 850 million people affected globally, regardless of age [13]. Disadvantaged populations are particularly vulnerable to the disease. The high costs of treatment and the significant health consequences associated with kidney disease contribute to its devastating effects. In Taiwan, CKD has become a major concern, ranking as the 9th leading cause of death over the past decade [14]. Research estimates that the national prevalence of CKD in Taiwan is approximately 11.9 %, affecting more than 2.5 million people [15,16]. Furthermore, Taiwan has a more than 1.5-fold greater prevalence of end-stage renal disease (ESRD) than the United States, with approximately 3400 individuals per 1 million in the general population experiencing kidney failure [17]. These statistics highlight the urgent need for improved access to affordable treatment options and increase awareness about CKD worldwide.

In recent years, cell-based therapies have garnered attention across various medical research fields. Mesenchymal stromal cells (MSCs) are extracted from diverse adult tissues, including adipose tissue, umbilical cord blood, bone marrow, and macrophage [18] MSCs are multipotent cells that can differentiate into tissues derived from mesoderm and have the ability to self-renew [19]. Furthermore, MSC possesses the ability to evade alloantigen recognition owing to their low immunogenicity and lack of expression of co-stimulatory molecules. The inherent immunomodulatory properties of MSCs, coupled with their minimal potential side effects, present a therapeutic alternative in this regard [20,21]. Recent studies suggest that administration of MSC-derived EVs ameliorates CKD in preclinical models [[22], [23], [24], [25]].

In the realm of renal diseases, EXO derived from MSC have gained significant attention due to their pathophysiological, diagnostic, and therapeutic roles [26]. These nanosized vesicles, ranging from 30 to 100 nm in diameter, originate from multivesicular bodies [27]. Under both physiological and pathological conditions, various cell release EXO into the blood or other bodily fluids, reflecting cellular responses to internal and external stimuli [28]. EXO carry a diverse cargo, including proteins, long noncoding RNAs, microRNAs (miRNAs), mRNAs, and lipids of particular note, miRNAs, which are noncoding, single-stranded small RNAs, which play a vital part in regulating gene expression by binding to the 3′ untranslated regions (UTRs) of target mRNAs, which leads to disintegration or translational suppression. [29,30]. Preclinical studies have showcased the positive impacts of MSC-derived EVs ameliorates CKD derived from cells, including secreted growth factors, microvesicles, and EXO, in models of chronic kidney injury [31]. These findings suggest a regenerative influence of cell-based therapies on renal function. Additionally, MSCs are actively employed in several clinical trials involving kidney transplant recipients, with the goal of enhancing immunosuppression and promoting improved regeneration [32]. Additionally, these EXO are free from the adverse aspects related to tumorigenic and immunogenic associated with cellular therapies, making them a safer and more viable avenue for future regenerative medicine [33].

In this meta-analysis and systematic review aimed to assess the impact of MSC derived EXO on outcome parameters related to chronic kidney disease function and morphology, scrutinizing both cell- and model-related aspects. The existing studies can enhance the design of future clinical investigations. Additionally, the insights gained can be utilized to refine current experimental animal models and interventions, thereby improving the quality of preclinical research in the future.

## 2 Result

### 2.1 Study selection and characteristics

**Fig. 1:**
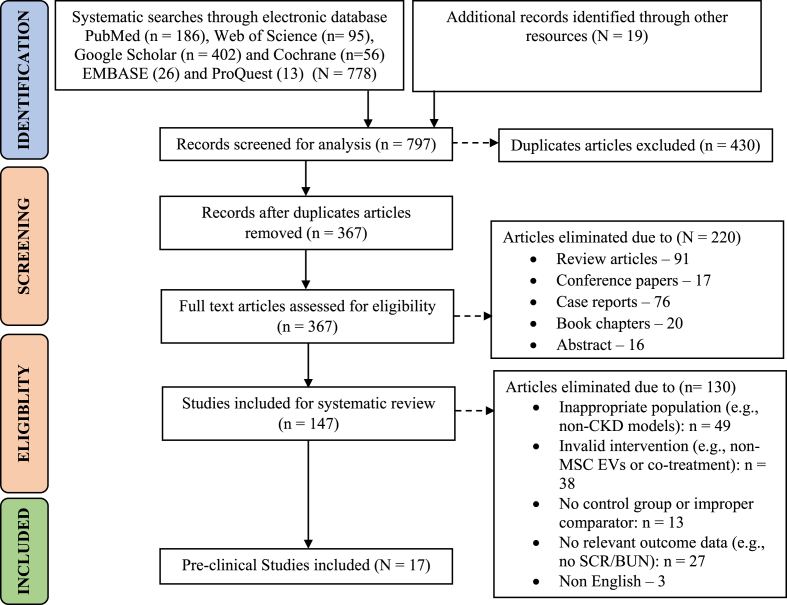
Study selection process according to PRISMA.

A total of 758 papers published from 2019 to 2023 were systematically categorized using esteemed databases, including PubMed (n = 186), Web of Science (n = 95), Google Scholar (n = 402), Cochrane (n = 56), EMBASE (n = 26) and ProQuest (n = 13) along with 19 additional records sourced from other sources. Out of these categorized articles 430 duplicate articles were removed, from the selection of these, 220 articles articles were excluded based on predefined eligibility criteria, including review articles (n = 91), conference papers (n = 17), case reports (n = 76), book chapters (n = 20), and abstracts (n = 16). Our meticulous screening process meticulously refined the dataset to 147 studies suitable for our systematic review. This subset underwent further scrutiny, resulting in the exclusion of 130 articles. These exclusions were attributed to diverse reasons, including publications: inappropriate population (e.g., non-CKD models) (n = 49), invalid intervention (e.g., non-MSC EVs or co-treatment) (n = 38), no control group or improper comparator (n = 13), no relevant outcome data (e.g., no SCR/BUN) (n = 27), non-English (n = 3). This exhaustive evaluation ultimately led to the inclusion of 17 pre-clinical studies [24,[34], [35], [36], [37], [38], [39], [40], [41], [42], [43], [44], [45], [46], [47], [48], [49]] that met our stringent inclusion criteria. The study selection process adhered to the PRISMA [50] flow diagram, as shown in Fig. 1.

### 2.2 Quality assessment

**Fig. 2:**
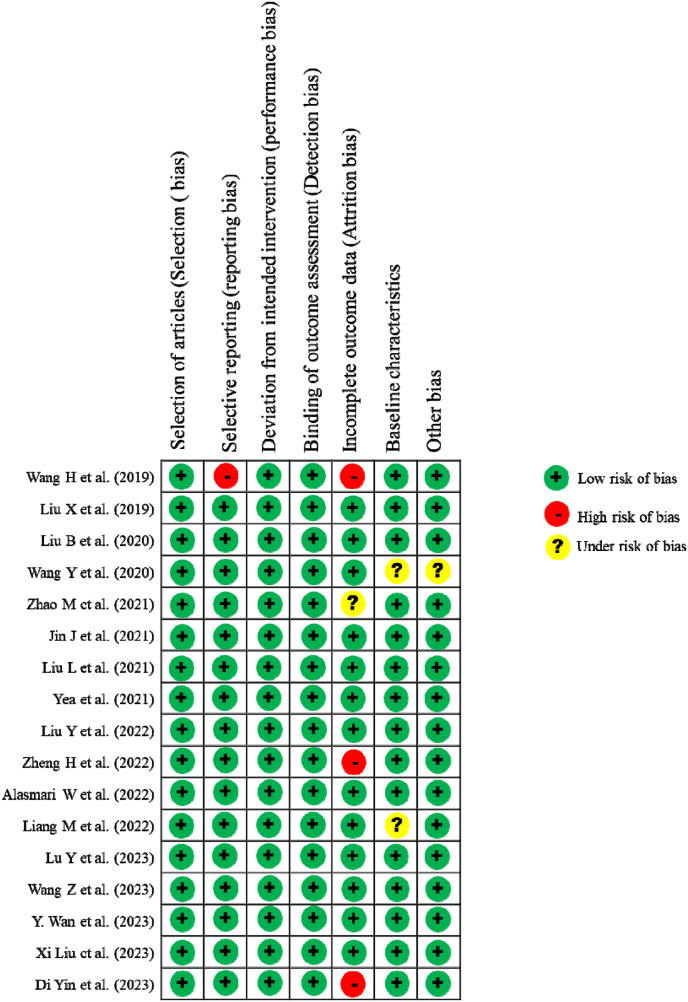
Risk of bias.

The risk of bias assessment for the studies included reveals variations in specific domains. Xi Liu et al. [43], Yingjie Liu et al. [36] and H. Wang et al. [49] is deemed to have a high risk of incomplete outcome data (attrition bias), indicating potential concerns regarding the completeness of outcome data reported in this study. Conversely, M. Liang et al. [38], Yan Wang et al. [24], H. Wang et al. [49] and Zhao M et al. [39] are characterized by a low risk of baseline characteristics and other bias, suggesting that the methods used to assess and measure outcomes in these studies are robust and unlikely to introduce bias, as shown in Fig. 2.

In accordance with MISEV2018 guidelines, we evaluated exosome characterization across included studies based on the presence of both exosome surface tetraspanins (e.g., CD63, CD81, CD9) and endosomal origin markers (e.g., TSG101, Alix). Out of 17 studies, 11 fully met the criteria, while six studies lacked one or more recommended markers. The studies included, H. Wang et al. [49], J. Jin et al. [47], and Ji H. Yea et al. [41], among others, which failed to report either surface or endosomal markers. Y. Wang et al. [24], and M. Zhao et al. [39], have been flagged showed methodological bias in our risk of bias assessment. We recommend that future investigations consistently adhere to MISEV guidelines to ensure the purity and identity of exosome preparations.

## 3 General characteristics

### 3.1 Clinical

CKD is a multifaceted and advancing health issue, often requiring a range of treatments and approaches. One area of promising exploration involves EXO, which has emerged for their potential therapeutic impact on CKD. To determine the efficacy and safety of employing EXO-based therapies for CKD, numerous clinical trials have been carried out. These trials encompass a spectrum of EXO sourced from different origins, such as MSCs, bone marrow, adipose tissue, umbilical cord, and various other cell types. The diversity in exosome sources is aimed at understanding their distinct compositions and potential roles in alleviating CKD-related complications.

To the best of our current knowledge, no clinical trials currently investigate MSC-derived exosomes for CKD treatment, we included registered trials employing whole MSC therapy to provide contextual insight into clinical translation trends. These trials exhibit a varied landscape in terms of clinical status, phases, locations, study types, sources of EXO, and study durations. Among the six trials with known clinical status, one has been completed (NCT02166489), four are actively recruiting (NCT05512988, NCT03939741), and NCT05362786 is active but not recruiting, while the status of the remaining two is unknown (NCT03321942, NCT05042206). These trials encompass both allogeneic and autologous approaches, with three employing allogeneic MSCs, three using autologous MSCs, and one unspecified. In terms of phases, three trials are in Phase I, two are in Phase I/II, and one does not specify its phase. Geographically, the trials are spread across different countries, including one in the United States, China, Iran, Korea, and Bangladesh. The MSC used in these trials primarily originates from bone marrow (n = 3), followed by adipose tissue (n = 2) and umbilical cord (n = 1), as summarized in Table 1. These trials were not included in the meta-analysis and are clearly labeled in Table 1 as exploratory evidence. Given the absence of exosome-specific trials, we have removed conclusions directly comparing MSC-EXOs and MSCs in terms of clinical safety. We also emphasize the need for future trials evaluating purified MSC-EXOs in CKD patients to validate preclinical efficacy and assess pharmacokinetics, dosing, and safety profiles in human subjects.

**Table 1:** Clinical trials involving MSCs – a contextual background.

S. No	Id no.	Location	Source	Autologous/allogeneic	Study type	Phase	Number of patients	Administration frequency	Start year	End year	Status	Outcome measures
1.	NCT05362786	United States	Bone marrow-derived mesenchymal stem cell	Allogenic	Non-randomized, open label	Phase I	14	I.V. injection, delivered MSC in one of two fixed dosing regimens at two time points	2022	N/A	Active, not recruiting	- Primary: adverse events and/or serious adverse events and change in Gfr value
2.	NCT05512988	China	Umbilical cord derived mesenchymal stem cells (UC-MSC)	Autologous	Randomized, double-blind, controlled clinical study	Phase I phase II	44	IV injection, each trial group received two injections throughout the entire course, with a cumulative dose of 2 × 10^6^ cells/kg/person.	2021	N/A	Recruiting	N/A
3.	NCT03321942	China	Adipose-derived mesenchymal stem/stromal cells (MSC)	N/A	Randomized, open label	N/A	100	Intravenous injection for 3 months	2017	N/A	Unknown status	- Primary: mass formation & creatinin - Secondary: Gfr
4.	NCT02166489	Iran	Bone marrow-derived mesenchymal stem cell	Autologous	Open label	Phase I	6	I.V. injection of high doses (2 × 10^6^) of autologous MSC per Kg of their body weight, sourced from bone marrow biopsies.	2014	2016	Completed	- Primary: mass formation - Secondary: Gfr
5.	NCT05042206	Korea	Allogeneic bone marrow derived mesenchymal stem	Allogeneic	Open label, single center	Phase I	10	I.V. injection of 10 ml dose 3 times in 2 weeks.	2021	N/A	Unknown status	- Primary: adverse event - Secondary: changes in EGfr, bun and creatinine
6.	NCT03939741	Bangladesh	Adipose derived stem cell (ADSC)	Autologous	Randomized, double-blind, controlled, open label	Phase I/phase II	31	I.V. injection of 5 ml stromal vascular fraction (SVF) containing ADSC.	2019	N/A	Recruiting	- Primary: change of Gfr & adverse events, - Secondary: Iga, igg, igm, c3, c4, crp, il-6, peripheral hemolymocyte subsets.

*Abbreviations:* N/A: not applicable; I.V.: intravenous.

### 3.2 Pre-clinical

This meta-analysis and systematic review meticulously explore exosome-based therapeutic interventions for chronic kidney disease (CKD) through a comprehensive analysis of diverse sources. The study encompasses data from various animal models, including C57BL/6 (n = 9), SD rats (n = 4), BALB/c (n = 2), Albino rats (n = 1), and Bred Fisher rats (n = 1), originating from countries such as the USA (n = 2), China (n = 13), Saudi Arabia (n = 1), and South Korea (n = 1). The investigation includes a gender-specific breakdown, focusing on human (n = 11) and animal (n = 17) subjects, and incorporates a variety of cell types, such as human umbilical cord MSC (hucMSCs) (n = 3), human bone marrow-derived MSCs (BM-MSC) (n = 4), adipose tissue-derived MSC (n = 1), among others. Diverse exosome isolation methods are examined, comprising ultracentrifugation (n = 8), centrifugation + filtration (n = 7), and exosome isolation kits (n = 2). Characterization techniques involve nanoparticle tracking analysis (NTA) (n = 2), transmission electron microscopy (TEM) (n = 5), both TEM and NTA (n = 9), and N/A (n = 1). The study identifies a consistent EXO size range of 30–150 nm, with common markers CD9, CD63, and TSG101. Administration routes vary, including intravenous (n = 14), intramuscular (n = 1), intraperitoneal (n = 1), and intracellular (n = 1), with concentrations ranging from 20 mg to 250 μg, providing a thorough overview of EXO -based therapies in managing CKD. At the culmination of this analysis, these findings underscore the diverse landscape of EXO -based therapies in addressing CKD, offering valuable insights into the potential efficacy and methodologies employed across various studies, aiming to elucidate optimal strategies for leveraging EXO therapy in CKD management, as summarized in Table 2.

**Table 2:** Characteristics of included preclinical studies for meta-analysis.

Author's name	Country	Year	Animal Model	Sex	Human/Animal	Exosome source	Passage number	Characterization	Isolation method	Exosome size	Exosome marker	Endosomal origin markers	Induced method	Concentration	Exosome admin.	End point	Outcome	Ref.
Wang H et al.	USA	2019	C57BL/6 mice	N/A	Animal	Primary mouse satellite cells	P2	NTA	Centrifugation + Filtration	91 ± 1.9 nm		Tsg101	I.M.	N/A	3rd day	14 days after injection	Exo/miR29 improves skeletal muscle atrophy and reduces kidney fibrosis by suppressing YY1 and TGF-b pathway proteins.	[49]
Liu X et al.	USA	2019	C57BL/6 mice	Male	Human	HKC-8	N/A	TEM	Ultracentrifugation	30–100 nm	CD63	TSG101	I.V.	20 mg/kg	1st day	11th day after injection	Tubule-derived exosomes contribute to renal fibrogenesis by transporting Sonic Hedgehog (Shh) ligand.	[45]
Liu B et al.	China	2020	SD rats	Male	Human	hUCMSCs	P3	TEM	Centrifugation + Filtration	50–100 nm	CD9, CD63 & CD81	NA	I.C.	200 μg	N.A.	14th day after injection.	HucMSC-Ex attenuated UUO-induced renal fibrosis and tubular cell apoptosis by inhibiting the ROS-mediated p38MAPK/ERK signaling pathway.	[44]
Wang Y et al.	China	2020	Bred Fisher rats	N/A	Animal	BM-MSCs	P2	N/A	Centrifugation + Filtration	N/A	CD44, CD29	Alpha 4-integrin	I.V.	N/A	0 day	14th day after injection.	MiR-294/miR-133 overexpression prevented TGF-β1-induced epithelial-mesenchymal transition in HK2 cells by inhibiting SMAD2/3 and ERK1/2 phosphorylation.	[24]
Zhao M et al.	China	2021	SD Rats	N/A	Animal	Rat urine sample	N/A	TEM, NTA	Ultracentrifugation	30–150 nm	CD63, CD81	NA	I.V.	12.50 g/kg	N.A.	18weeks after injection	MHCD mitigated renal fibrosis in IgA nephropathy rats by suppressing the TGF-β1/Smad3 pathway through exosomal downregulation of TGF-β1 expression.	[39]
Jin J et al.	China	2021	C57BL/6 mice	Female	Human	BM-MSCs	N/A	TEM, NTA	Exosome Isolation Kit	50–150 nm	CD 9, CD 81	NA	I.V.	50 μg	Twice a week	28th day	MSC exosomal anti-let-7i-5p combats TGF-β1-induced fibrosis in NRK52E cells and UUO-induced renal fibrosis in vivo.	[47]
Liu L et al.	China	2021	C57BL/6 mice	Male	Human	PSC-MSCs	P3∼8	NTA	Centrifugation + Filtration	10∼150 nm	CD90, CD45, CD105 & CD19	NA	I.V.	1 × 10^11^ (Particle amount)	N.A.	14th day after injection.	Upregulating SIRT6 expression while downregulating β-catenin and its downstream products.	[48]
Liu L et al.	S. Korea	2021	BALB/c mice	Male	Human	AD-MSC	P2	TEM, NTA	Exosome isolation kit	173.02 ± 2.34 nm	CD81 & CD9	NA	I.V.	100 μg	twice a week for 2 weeks	3rd week after administration	Exocue treatment downregulated CKD-related miRNAs, improving kidney function via aquaporin upregulation and reduced urea and creatinine levels.	[41]
Liu Y et al.	China	2022	SD rats	Male	Human	BM-MSCs	P4∼6	TEM, NTA	Centrifugation + Filtration	30–150 nm	CD9, CD63, & CD81	NA	I.V.	150 μg/week	Once a week	16th week	BMSC-Exo strengthened si-Smurf2's protective effect against TGF-β1-induced fibrosis.	[36]
Zheng H et al.	China	2022	C57BL/6 mice	Male	Human	293 cell line human embryonic kidney cells	N/A	TEM	Ultracentrifugation	100 nm	CD63, CD81	NA	I.V.	100 μg	Once a week	28th day	Exo-miR-26a relieved kidney-downregulated miR-26a expression, tubular injury, and aldosterone (ALD)-induced TIF.	[40]
Alasmari W et al.	Saudi Arabia	2022	Albino rats	Female	Animal	BM-MSCs	P3	TEM	Centrifugation + Filtration	70 nm	CD63, CD81	NA	I.V.	100 μg	24 h after ovariectomy	8th week	Exosomes hinder CKD progression by lowering gene expression of NGAL, TGF-β1, and α-SMA.	[37]
Liang M et al.	China	2022	C57BL/6 mice	N/A	Human	HK-2 cells proximal tubular cell line	N/A	TEM, NTA	Centrifugation + Filtration	100–150 nm	CD63	TSG101	I.V.	N/A	twice weekly for 4 weeks	28th day	MiR-374a-5p hinders renal fibrosis progression by modulating the MAPK6/MK5/YAP axis.	[38]
Lu Y et al.	China	2023	BALB/c mice	Male	Animal	Mouse renal tubular cell line TCMK-1	N/A	TEM	Ultracentrifugation	145.8 nm	HSP70, CD63	TSG101	I.V.	100 μg	Per day for 5 days	7th day	TGF-β-induced TECs co-cultured with macrophages led to M1 polarization. Exosomes from TECs without TGF-β or with TGF-β alone did not induce M1 macrophage markers.	[34]
Wang Z et al.	China	2023	C57BL/6 mice	Male	Human	hucMSCs	N/A	TEM, NTA	Ultracentrifugation	30–150 nm	CD63,	Tsg101, & Alix	I.V.	100 μg	Once 7 days after surgery	14th day	Inhibited expression of proteins related to the Wnt/β-catenin signaling pathway.	[35]
Y. Wan et al.	China	2023	SD Rats	Male	Human	hucMSCs	P5	TEM, NTA	Ultracentrifugation	30–150 nm	CD63	Tsg101, & Alix	I.V.	250 μg	Immediately after reperfusion	24 h after injection	HucMSC-Ex reduced pyroptosis-related proteins (NLRP3, GSDMD, caspase-1, and IL-1β) in renal tissue of IRI rats.	[46]
Xi Liu et al.	China	2023	C57BL/6 mice	Male	Human	HKC-8	N/A	TEM, NTA	Ultracentrifugation	30–150 nm	CD63	Tsg101	I.V.	200 μg	Once in 2 days	14th day	Exo-TNFAIP8 promotes fibroblast survival by inducing p53 degradation and stimulating proliferation.	[43]
Di Yin et al.	China	2023	C57BL/6 mice	Male	Animal	Tubular epithelial cells of mouse	N/A	TEM, NTA	Ultracentrifugation	129.5 ± 8.2 nm	CD63, CD9	Tsg101, & Alix	I.P.	200 μg	Once on day 1	3rd day	Quercetin inhibits exosome release, exerting renoprotective effects by suppressing Hsp70 or Hsp90.	[42]

*Abbreviations:* N/A: not applicable; I.V.: intravenous; IM: Intramuscular; I.C: Intracellular; IP: Intraparietal; HKC-8:Human proximal tubular epithelial cells; HbMSCs: Human bone marrow derived; hucMSCs: Human umbilical cord mesenchymal stem cells; AD: Human adipose; PSC-MSCs: pluripotent stem cell-derived mesenchymal stem cells.

### 3.3 Meta-analysis

A. Primary outcome

i. Blood urea nitrogen (BUN)

**Fig. 3:**
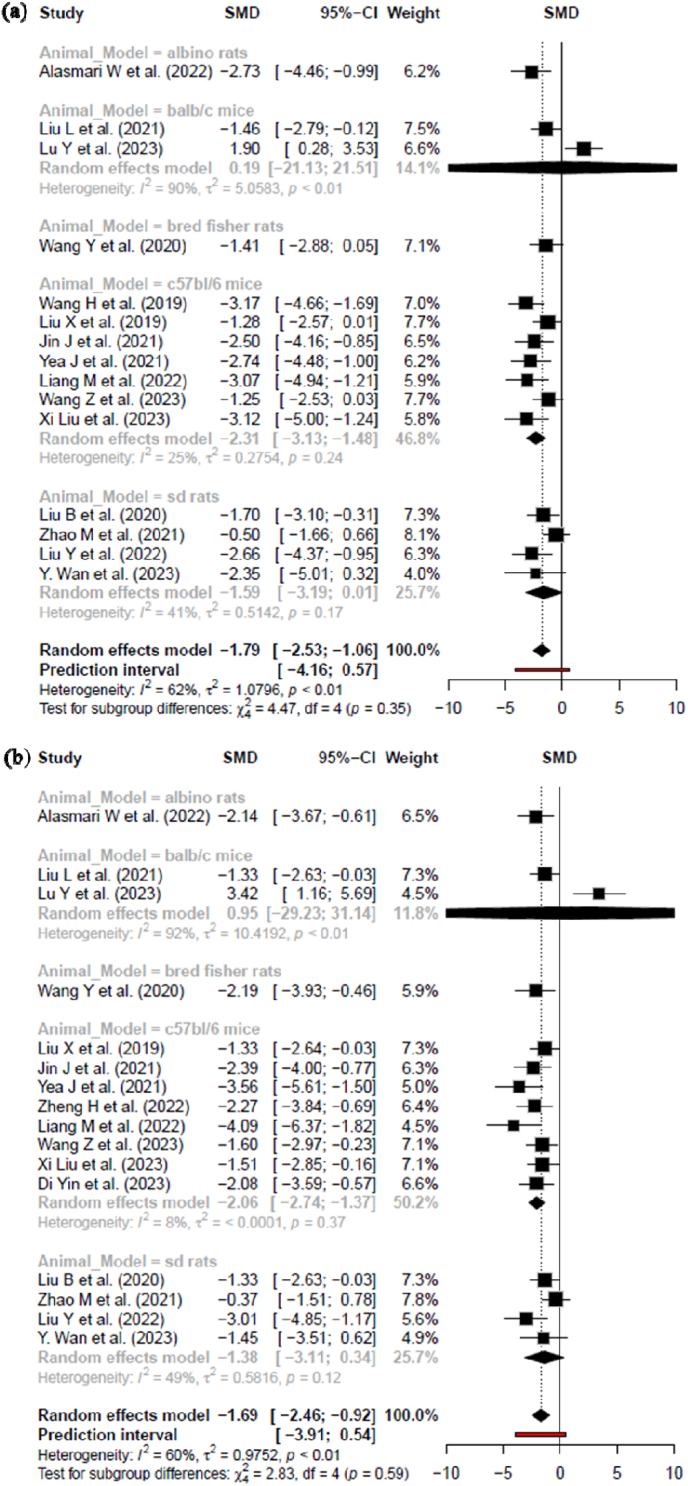
Forest plots of standardized mean differences (SMDs) with 95 % confidence intervals (CIs) for (A) BUN and (B) SCR levels following MSC-derived exosome treatment in CKD models. Statistical analysis was performed using a random-effects model, between-study heterogeneity was quantified using I^2^, τ^2^, and Cochran's Q test p-values. Effect sizes are shown for individual studies and pooled estimates. Negative SMD values indicate a reduction in renal biomarkers.

BUN is a waste product that is produced when protein is broken down in the body [51]. The analysis of BUN in CKD reveals a moderate degree of heterogeneity, as indicated by an I^2^ value of 62 %, τ^2^ of 1.0796 %, and a significant p-value of less than (*p* < 0.01). This substantial heterogeneity underscores the diversity in BUN outcomes across the studies included in the review. The calculated standard mean deviation (SMD) and its 95 % confidence interval (CI) further emphasize this variability, with a value of −1.79 [−2.53; −1.06] of random effects model as depicted in Fig. 3A.

ii. Serum creatinine (SCR)

SCR is a waste product that is produced when creatinine, a chemical that is found in muscle tissue, is broken down [51]. In the context of CKD, the analysis of SCR levels reveals a moderate degree of heterogeneity, as evidenced by an I^2^ value of 60 %, τ^2^ of 0.9752 %, and a significant p-value of less than 0.01 (p < 0.01). This substantial heterogeneity underscores the varied outcomes observed across studies included in the analysis. The calculated proportion and its 95 % CI further emphasize this diversity, with the value of −1.69 [−2.46; −0.92] (of Random effects model), as depicted in Fig. 3B.

B.Secondary outcome

i. Animal model subgroup analysis based on BUN level

**Fig. 4:**
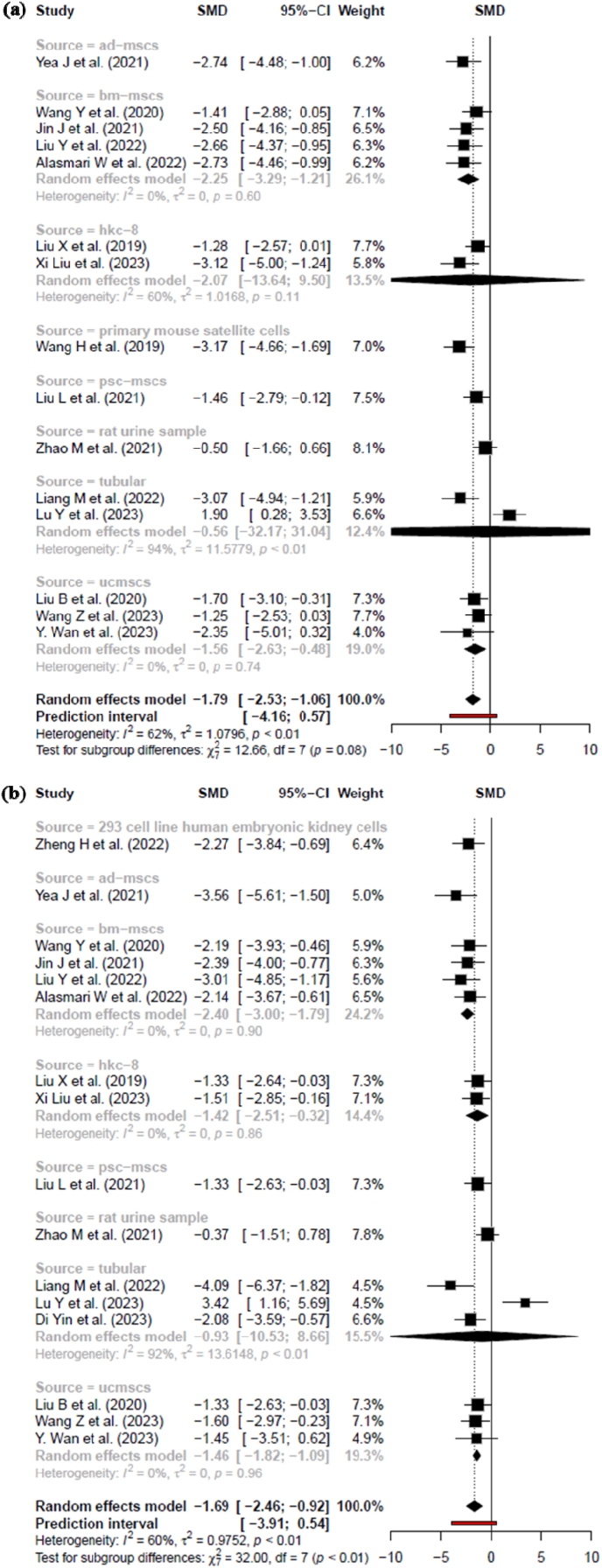
Subgroup analysis based on animal model with their 95 % confidence intervals (A) efficacy in BUN reduction of MSC derived EXOs (B) efficacy in SCR reduction of MSC derived EXOs. Study heterogeneity was assessed using I^2^, τ^2^, and Cochran's Q test. Test for subgroup differences (χ^2^ and p-values) are reported. Negative SMD values indicate a reduction in renal biomarkers, suggesting therapeutic benefit.

To evaluate the therapeutic impact of exosome treatment on blood urea nitrogen (BUN) levels in preclinical models of chronic kidney disease (CKD), a subgroup analysis was conducted across different animal models. The analysis revealed varying degrees of effect and heterogeneity among species (Fig. 4A).

In the C57BL/6 mouse subgroup, a consistent and statistically significant reduction in BUN levels was observed, with a pooled standardized mean difference (SMD) of −2.31 [−3.13, −1.48]. This subgroup contributed the largest proportion to the overall meta-analysis (46.8 %) and demonstrated low heterogeneity (I^2^ = 25 %, τ^2^ = 0.2754, p = 0.24). Conversely, the BALB/c mice subgroup exhibited extreme heterogeneity (I^2^ = 90 %, τ^2^ = 5.0583, p < 0.01), with a pooled estimate of 0.19 [−21.13, 21.51]. The wide confidence interval and instability of this estimate, driven by opposing effect directions in just two included studies, suggest caution in interpretation. This subgroup accounted for 14.1 % of the overall weight. Among rat models, the Sprague Dawley (SD) rats showed a moderate effect size (SMD = −1.59 [−3.19, 0.01]) with moderate heterogeneity (I^2^ = 41 %, τ^2^ = 0.5142, p = 0.17), contributing 25.7 % to the total analysis. The Bred Fisher rat subgroup, represented by a single study, showed an SMD of −1.41 [−2.88, 0.05] and contributed 7.1 % weight. Similarly, the Albino rat subgroup (also single-study) showed a more pronounced effect (SMD = −2.73 [−4.46, −0.99], weight = 6.2 %).

Overall, the pooled estimate across all models indicated a significant reduction in BUN following exosome treatment (SMD = −1.79 [−2.53, −1.06]), despite moderate heterogeneity across studies (I^2^ = 62 %, τ^2^ = 1.0796, p < 0.01). The prediction interval [−4.16, 0.57] highlights the variability in potential future outcomes. Importantly, the test for subgroup differences was not statistically significant (χ^2^ = 4.47, df = 4, p = 0.35), indicating no conclusive evidence of effect modification by animal model type.

ii. Animal model subgroup analysis based on SCR level

In the investigation of SCR levels across various animal models of CKD, significant heterogeneity is observed. In the C57BL/6 mouse subgroup, a robust pooled effect was observed (SMD = −2.06 [−2.74, −1.37]), indicating a consistent decrease in SCR. Despite the large number of included studies, moderate heterogeneity was present (I^2^ = 48 %, τ^2^ = 0.1226, p = 0.037), and this subgroup contributed the largest weight to the overall analysis (50.2 %). The Sprague Dawley (SD) rat subgroup showed a comparable effect size (SMD = −1.34 [−2.57, −0.11]) with moderate heterogeneity (I^2^ = 49 %, τ^2^ = 0.1214, p = 0.12), contributing 25.7 % to the total weight. These findings support the utility of SD rats as a reliable preclinical model in evaluating exosome-based therapies for renal dysfunction. In the Bred Fisher rat model, represented by a single study, a substantial reduction in SCR was also detected (SMD = −2.19 [−3.93, −0.46]), contributing 5.9 % to the meta-analysis. The BALB/c mouse subgroup displayed extreme between-study heterogeneity (I^2^ = 92 %, τ^2^ = 10.4192, p < 0.01) and a non-significant pooled estimate (SMD = 0.95 [−29.23, 31.14]), largely driven by the inclusion of two studies with highly divergent effect directions. This subgroup contributed 11.8 % to the overall weight but demonstrated poor estimate stability, warranting cautious interpretation. The Albino rat subgroup exhibited a pronounced reduction in SCR (SMD = −2.14 [−3.67, −0.61]), contributing 6.5 % to the analysis. This consistent and significant effect may reflect a strong treatment response in this strain.

Across all animal models, the pooled random-effects estimate showed a significant overall benefit of exosome therapy in reducing SCR levels (SMD = −1.69 [−2.46, −0.92]), with moderate heterogeneity (I^2^ = 60 %, τ^2^ = 0.09752, p < 0.01). The prediction interval (−3.91 to 0.54) reflects the expected range of effects in future studies. Notably, the test for subgroup differences was not statistically significant (χ^2^ = 2.83, df = 4, p = 0.59), suggesting that the treatment effect was generally consistent across species, despite some subgroup-specific variability (Fig. 4B).

iii. Subgroup Analysis Based on the Source of Exosomes on BUN level

A subgroup meta-analysis was conducted to investigate whether the therapeutic efficacy of exosome treatment in reducing disease severity varied according to the cellular source of exosomes. The analysis included exosomes derived from eight distinct sources, revealing variation in effect sizes across subgroups. Notably, exosomes from adipose-derived mesenchymal stem cells (AD-MSCs) showed a strong effect (SMD = −2.74 [−4.48, −1.00]), as did those from bone marrow-derived MSCs (BM-MSCs) (SMD = −2.25 [−3.29, −1.21]), both suggesting robust reductions in pathological markers. Similarly, primary mouse satellite cell-derived exosomes exhibited a considerable effect (SMD = −3.17 [−4.66, −1.69]). On the other hand, exosomes from human kidney epithelial cells (HKC-8) and urine-derived vesicles displayed smaller or non-significant effects, with SMDs of −2.07 [−13.64, 9.50] and −0.50 [−1.66, 0.66], respectively, reflecting either limited efficacy or study-specific variability.

**Fig. 5:**
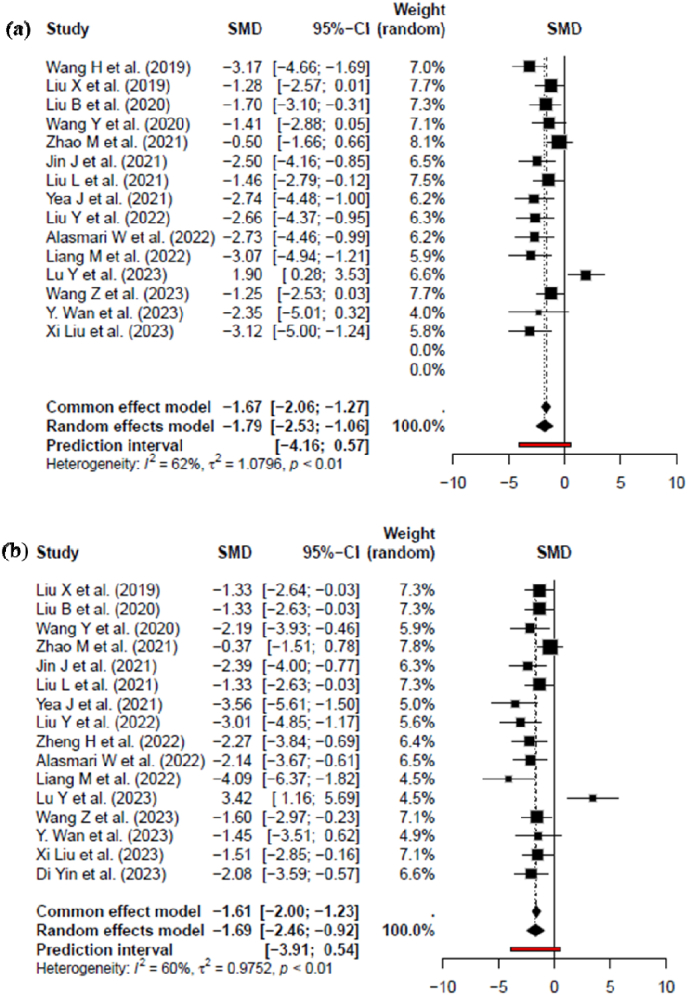
Subgroup analysis based on source of exosome model with their 95 % confidence intervals (A) efficacy in BUN reduction of MSC derived EXOs (B) efficacy in SCR reduction of MSC derived EXOs. Study heterogeneity within subgroups was quantified using I^2^, τ^2^, and Q statistics. A test for subgroup differences (χ^2^ and p-values) was performed to evaluate whether treatment efficacy varied significantly by exosome origin. Negative SMD values indicate therapeutic benefit through reduction in renal injury markers.

Interestingly, tubular cell-derived exosomes showed the highest degree of heterogeneity (I^2^ = 94 %), with highly divergent results between two studies (SMD = −3.07 vs. +1.90), highlighting inconsistency in therapeutic outcomes. Exosomes from umbilical cord MSCs (UCMSCs) and pluripotent stem cell-derived MSCs (PSC-MSCs) also showed beneficial effects with SMDs of −1.56 and −1.46, respectively. The overall test for subgroup differences approached statistical significance (χ^2^ = 12.66, df = 7, p = 0.08), indicating a potential trend toward source-dependent efficacy that warrants further investigation. Despite moderate overall heterogeneity (I^2^ = 62 %), the global effect remained statistically significant (SMD = −1.79 [−2.53, −1.06]), reinforcing the therapeutic potential of exosomes regardless of origin, while emphasizing the need for source standardization in translational applications (Fig. 5A).

iv. Subgroup Analysis Based on the Source of Exosomes on SCR level

To determine whether the source of exosomes influenced their therapeutic efficacy in experimental models with SCR level, a subgroup analysis was conducted across eight distinct cell origins. Among these, bone marrow-derived mesenchymal stem cell (BM-MSC) exosomes exhibited a consistent and substantial effect on outcome measures (SMD = −2.40 [−3.00, −1.79]), with no observed heterogeneity (I^2^ = 0 %, τ^2^ = 0, p = 0.90), and constituted the largest subgroup by weight (24.2 %). Similarly, umbilical cord MSC (UCMSC)-derived exosomes demonstrated robust effects (SMD = −1.46 [−1.95, −0.98], I^2^ = 0 %) across three studies, contributing 19.3 % to the total meta-analysis. Human kidney epithelial cell-derived exosomes (HKC-8) and pluripotent stem cell-derived MSC (PSC-MSC) exosomes yielded comparable, moderate treatment effects (SMD = −1.42 and −1.33, respectively), both with low heterogeneity.

In contrast, exosomes from rat urine exhibited a negligible and non-significant effect (SMD = −0.37 [−1.51, 0.78]), suggesting limited utility in this model. Notably, the tubular cell-derived exosome subgroup displayed extreme heterogeneity (I^2^ = 92 %, τ^2^ = 13.6148, p < 0.01) with highly divergent results between studies, yielding a pooled SMD of −2.94 [−6.50, 0.61]. This suggests variability in either experimental design or exosome functionality within this cell type. Exosomes derived from AD-MSCs (SMD = −3.56 [−5.61, −1.50]) and 293 human embryonic kidney cells (SMD = −2.27 [−3.84, −0.69]) also showed strong individual effects but were each represented by only one study.

The overall pooled effect across all subgroups remained statistically significant (SMD = −1.69 [−2.46, −0.92]), indicating the general efficacy of exosome therapy. However, the test for subgroup differences was highly significant (χ^2^ = 32.00, df = 7, p < 0.01), underscoring that the therapeutic effect is substantially influenced by the cellular origin of exosomes. These findings emphasize the critical importance of exosome source selection in both experimental design and translational development (Fig. 5B).

### 3.4 Meta-regression and heterogeneity analysis

To investigate the sources of heterogeneity observed in the pooled effect sizes, meta-regression analyses were conducted using two key moderators: the type of animal model employed and the cellular origin of the MSC-derived exosomes. These variables were selected based on their known influence on experimental outcomes in preclinical studies of CKD.

The meta-regression based on animal model type revealed significant variability in the therapeutic response to exosome treatment. Rodent models such as rats and mice, though commonly used, displayed differing sensitivities to MSC-derived exosomes. Certain models demonstrated more pronounced reductions in BUN and SCR levels following treatment, as visualized in Supplementary Figure 1. These differences suggest that the underlying pathophysiology modeled in each animal system may affect responsiveness to regenerative interventions and must be considered when translating findings to clinical contexts. In parallel, meta-regression based on the source of MSC-derived exosomes also showed substantial variation in treatment efficacy. Exosomes derived from different tissue sources—such as (BM-MSCs, AD-MSC, and UC-MSCs yielded varying degrees of renal function improvement. For example, exosomes from UC-MSCs tended to show a higher magnitude of effect in reducing renal injury markers, suggesting a potentially enhanced immunomodulatory or reparative profile. These findings, illustrated in Supplementary Figure 2, support existing literature proposing that the regenerative potential of MSC-exosomes is influenced by the tissue of origin, which may affect exosomal cargo composition and bioactivity.

In addition, funnel plot analyses were conducted for each moderator subgroup (animal model and exosome source) to assess the risk of publication bias. The plots (Supplementary Figs. 3 and 4) showed relatively symmetrical distributions, indicating an acceptable level of publication bias across the included studies.

## 4 Discussion

In this comprehensive systematic review and meta-analysis, encompassing 17 preclinical and 6 clinical studies, we provide a thorough examination of the impact of MSC-EXOs on diverse models of CKD. Preclinical and clinical studies have demonstrated that treating patients with CKD using MSC-EXOs leads to reductions in SCR and BUN levels, improved glomerular filtration rate, and protection of renal functions, along with the suppression of inflammatory responses. Though our knowledge of the distinct parts of vesicular structures, their specific functions, and their roles as therapeutic vectors, biomarkers, and contributors to autoimmune disorders is still insufficient. The exploration of EXOs in CKD pathogenesis is still in its early stages, with limited research compared to other diseases. Further research is essential for the clinical application of MSC-EXOs. Although the safety of MSC-EXOs has been initially confirmed through animal experiments, extensive studies are required to ensure their safety for clinical use. Various MSC sources, such as bone marrow, adipose tissue and umbilical cord, contribute to EXOs derivation, necessitating additional experiments to assess the safety of different MSC- EXOs.

The delivery of EXOs has various benefits as a cell-free therapeutic approach, such as enhanced stability, reduced immunogenicity, permeability, and cytotoxicity [52]. As a result, employing EXOs could offer a practical and secure substitute for cell-based treatments. Many studies attribute these beneficial effects primarily to the RNA cargo carried by EVs, and it's noteworthy that these effects can be abrogated by RNase treatment. Numerous miRNA candidates, such as miR29, miR-294/miR-133, miR-26a, and miR-374a-5p, have been implicated in the pathophysiological processes of CKD. Research has revealed specific mechanisms through which various miRNAs contribute to mitigating kidney fibrosis and related complications in CKD. For instance, miR29 has been shown to ameliorate skeletal muscle atrophy and diminish kidney fibrosis by suppressing YY1 and proteins involved in the TGF-β pathway [49]. Additionally, by blocking the phosphorylation of SMAD2/3 and ERK1/2, miR-294/miR-133 has shown promise in preventing TGF-β1-induced epithelial-mesenchymal transition in HK2 cells [24]. Moreover, downregulation of miR-26a expression has been associated with reduced tubular injury and tubulointerstitial fibrosis induced by aldosterone [40]. Furthermore, miR-374a-5p has been shown to impede the progression of renal fibrosis by modulating the MAPK6/MK5/YAP axis, ultimately leading to reductions in urea and creatinine levels [38].

Given the heterogeneity of CKD induction models among the included studies, we further classified them based on the underlying pathophysiological mechanism: Obstructive models (e.g., Unilateral Ureteral Obstruction – UUO); Inflammatory models (e.g., Ischemia-Reperfusion Injury – IRI); Immune-mediated/metabolic models (e.g., IgA nephropathy, adenine-induced nephropathy). This classification reflects the different disease trajectories and cellular targets activated in CKD. Obstructive models primarily involve mechanical stress and extracellular matrix remodeling with limited systemic inflammation, whereas IRI models are characterized by oxidative stress, cytokine storms, and macrophage-driven fibrosis. In contrast, immune/metabolic models simulate chronic renal dysfunction under persistent immune dysregulation and fibrogenesis.

Our analysis reveals that MSC-derived exosomes (MSC-EXOs) exhibit therapeutic activity across all three model types, though the dominant mechanism of action appears model-dependent. For example, in obstructive models such as UUO, HucMSC-EXOs have been shown to reduce fibrosis and prevent tubular apoptosis via inhibition of the ROS-mediated p38MAPK/ERK pathway [39,44,45]. [47]. PSC-MSCs-derived EXO have shown the ability to upregulate SIRT6 expression while downregulating β-catenin and its downstream products [48]. Additionally, BM-MSC-derived EXO have enhanced the protective effects against TGF-β1-induced fibrosis when combined with si-Smurf2 [36]. BM-MSCs EXOs have further demonstrated their impact on hindering CKD progression by lowering the gene expression of NGAL, TGF-β1, and α-SMA [37]. A metabolic models like IgA nephropathy, modified EXOs with heparin-chitosan MHCD suppressed TGF-β1/Smad3 signaling and fibrogenesis [38]. Meanwhile, in inflammatory IRI models, HucMSC-EXOs decreased pyroptosis-related markers (e.g., IL-1β, NLRP3, caspase-1, GSDMD) and attenuated inflammation-mediated injury. [34,35]. These findings are supported by supplementary meta-regression and funnel plot analyses (Supplementary Figures 1–4), which provide additional insight into sources of heterogeneity and potential publication bias.

Additional studies support diverse mechanisms of MSC-EXO action, including miR-29-mediated ECM suppression [48], SIRT6 upregulation and β-catenin inhibition [47], and modulation of macrophage polarization [33]. EXOs also contributed to fibroblast survival through p53 degradation pathways [42,45], while agents like quercetin were found to inhibit EXO release and mitigate renal injury by targeting Hsp70/90 [24]. Taken together, these findings suggest that anti-fibrotic effects of MSC-EXOs (e.g., via miR-29, miR-374a-5p) are broadly applicable across obstructive and immune-mediated models, whereas anti-inflammatory and pyroptosis-inhibiting effects are more prominent in IRI models [43,46]. [42].

Understanding the specific study design and factors influencing the efficacy of EXOs based treatments can enhance the planning of future experimental studies and aid in designing studies for specific patient populations. Accordingly, for studies with available creatinine (SCR) and urea data (BUN), we conducted uni-variable stratified meta-analyses to explore potential predictors for EXO-based therapy effectiveness across diverse CKD settings. To elucidate the impact of EXO treatment on various animal models, we conducted subgroup analysis graphs. Our findings indicate that the functional efficacy of EXO therapy varies depending on the model employed. Notably, a significant proportion of the animal records analyzed in our study pertained to CKD models.

In evaluating the quality of included preclinical studies (Table 2), we identified several methodological gaps. Notably, the sex of the animals was reported in only 3 out of 17 studies. Considering that hormonal differences influence CKD progression—with male rodents, especially SD rats, showing greater susceptibility to progressive fibrosis—the omission of sex data introduces potential selection bias. Studies lacking this information (e.g., Haidong Wang, 2019) have been flagged as high risk in our bias assessment. To improve reproducibility and transparency in future research, we strongly advocate for adherence to the ARRIVE (Animal Research: Reporting of In Vivo Experiments) guidelines, which mandate clear reporting of sex, species, strain, and experimental conditions in animal studies. Standardizing these variables is essential for translating preclinical findings into clinically relevant interventions [53].

In addition to these reporting gaps, inconsistency in exosome characterization emerged as another significant limitation. Although we evaluated all included studies based on the MISEV2018 (Minimal Information for Studies of Extracellular Vesicles) criteria, some studies failed to report critical exosomal surface markers (CD63, CD81, CD9) or endosomal origin markers (TSG101, Alix). These markers are essential for validating the identity, purity, and source of extracellular vesicles, and for distinguishing exosomes from other vesicle subtypes or cellular debris. The absence of standardized exosome characterization increases the risk of experimental bias and may contribute to variability in therapeutic outcomes. We therefore advocate that future studies implement the MISEV2018 guidelines as a minimum reporting standard to ensure rigor, reproducibility, and confidence in exosome-based therapeutic research [54].

Although the preclinical evidence for the therapeutic efficacy of MSC-derived exosomes in CKD is compelling, there remains a substantial gap in clinical validation. To date, no completed clinical trials have specifically evaluated purified MSC-derived exosomes for CKD treatment. While several ongoing trials are investigating whole MSC-based therapies, it is inappropriate to extrapolate safety or efficacy conclusions to exosome-based interventions due to critical differences in pharmacokinetics, biodistribution, immunogenicity, and production scalability. As such, caution is warranted when interpreting clinical insights from MSC therapies in the context of exosome applications. There is an urgent need for early-phase (Phase I) clinical trials to assess the safety, biodistribution, optimal dosing strategies, and pharmacokinetic profiles of MSC-EXOs in human subjects. These foundational studies will be instrumental in determining the translational potential of exosome-based therapies and in informing regulatory pathways for their clinical development.

As a cell-free therapeutic approach, EXO present numerous advantages, characterized by high stability and permeability, as well as low immunogenicity and cytotoxicity [55]. This suggests that the administration of EXOs could serve as a viable and safe alternative to cell-based therapies. Additionally, substantial heterogeneity exists in the CKD models used across the selected studies. These include unilateral ureteral obstruction (UUO), ischemia-reperfusion injury (IRI), IgA nephropathy, and adenine-induced nephropathy, each of which invokes distinct pathophysiological mechanisms. For instance, UUO primarily drives mechanical obstruction-induced fibrosis, whereas IRI induces inflammatory and oxidative stress-mediated fibrosis. Recognizing these distinctions, we have now introduced a mechanism-based classification of studies in the Discussion, comparing obstructive vs. metabolic/inflammatory fibrosis models, to better contextualize the therapeutic actions of MSC-EXOs.

## 5 Materials and methods

a. Literature search

For this systematic review and meta-analysis, we conducted a comprehensive and structured literature search across multiple international databases, including PubMed/MEDLINE, Web of Science, Scopus, EMBASE, Google Scholar, CINAHL, LILACS, SciELO, and the Cochrane Central Register of Controlled Trials (CENTRAL). The search was designed to identify preclinical and clinical studies reporting the therapeutic effects of mesenchymal stem cell-derived exosomes (MSC-EXOs) in chronic kidney disease (CKD) The search period spanned from January 1, 2019, to January 31, 2024, and included articles published in English. The strategy integrated both Medical Subject Headings (MeSH) and free-text terms, using Boolean operators to maximize sensitivity. The primary search string was: (“extracellular vesicle” OR “EV” OR “exosome”) AND (“mesenchymal stem cell” OR “MSC” OR “stromal cell”) AND (“chronic kidney disease” OR “CKD” OR “renal fibrosis” OR “chronic renal failure” OR “chronic renal insufficiency” OR “kidney dysfunction”). We assessed study quality using defined criteria from the Collaborative Approach to Meta-analysis and Review of Animal Data in Experimental Studies (CAMRADES) risk of bias checklist. Moreover, for clinical studies, we conducted a thorough data search utilizing a comprehensive database encompassing privately and publicly funded clinical trials conducted globally, available at https://clinicaltrials.gov/. Additionally, manual searches of bibliographies and reference lists were performed to identify any additional relevant studies. No ethical approval was required as the meta-analysis relied solely on published articles.

b. Inclusion criteria

Following the elimination of duplicates, All studies were screened against predefined inclusion criteria based on the PICOS framework, including; a) Population: rodent models of CKD (UUO, IRI, IgA nephropathy, adenine diet, etc.) involved either allogeneic, xenogeneic or autologous approaches; b) Intervention: administration of extracellular vesicles derived exclusively from mesenchymal stem cells, preconditioned, and modified MSC-EXOs (such as those transfected with genes or featuring overexpression of proteins or microRNAs); c) Comparison: untreated or placebo-treated CKD animals; Outcomes: at least one renal outcome (e.g., BUN, SCR, GFR, fibrosis); d) Study Design: original preclinical or clinical research (English language only).

c. Exclusion criteria

Exclusion criteria involved: (1) studies lacking a control group or with non-randomized design; (2) use of interventions that included co-administration of other bioactive agents or stem cell-derived EVs not strictly isolated from MSCs; (3) outcomes not reporting at least one key renal biomarker (e.g., SCR, BUN, GFR, histological fibrosis score) (4) duplicate or overlapping data; (5) review articles, case reports, commentaries, conference abstracts, and book chapters; and non-English language articles.

d. Data extraction

Data extraction from all eligible studies encompassed gathering the following information for the clinical trials table: authors' names, study location, status of the trials, study year, type of kidney disease, study type, phase, patients' number, autologous/allogeneic, administration, frequency, outcome measures and intervention findings. Similarly, the preclinical trials table was compiled using the following details: author's name, country, publication year, animal model and their sex, human or animal derived, EXO source, isolation methods, modified strategy, and outcomes. Additionally, another data was compiled in a table by using information such as: author's name, characterization techniques, EXO size, induction methods, concentration, time of EXO administration and end point of the study. Sex-based reporting was performed for evaluating study quality, given known differences in CKD pathophysiology between male and female animals. Whenever available, the sex of experimental animals was extracted and included in the dataset. Studies failing to report animal sex were flagged during the risk of bias assessment. Spreadsheets were created to facilitate the extraction and synthesis of the data by using Excel® (Microsoft® Office Excel 2021) and subjected to pre-testing before complete extraction. Citations from the compiled papers were managed using Mendeley software (version 2.105.0, Elsevier, London, UK).

e. Quality assessment

Assessing publication bias is crucial to ensuring the integrity and credibility of the meta-analysis focused on the effects of EXOs on various aspects of CKD. To gauge the potential impact of publication bias in our findings, we applied several established techniques widely recommended in the field. A key method involved visually inspecting a bias risk graph for asymmetry, which may indicate the presence of publication bias. By employing these comprehensive approaches, our goal was to systematically address any potential bias and guarantee that our meta-analysis offers an unbiased synthesis of the current evidence regarding the positive effects of EXOs in the context of CKD.

f. Statistical analysis

The study compiled data on CKD, EXO source, and group sizes from papers or correspondence with authors. Employing random-effects models in meta-analysis, individual effects were considered, avoiding fixed effects to effectively address unobserved heterogeneity. Results were presented using effect size and 95 % CI. The metafor package in R (https://www.R-project.org/) facilitated all analyses. Notably, the meta-analysis specifically targeted SCR and BUN levels within the study.

## Ethics approval and consent to participate

Not applicable'.

## Consent for publication

All authors have given their consent to publish.

## Availability of data and materials

The authors confirm that the data supporting the findings of this study are available within the article.

## Authors' contributions

All authors have equally contributed to the conceptualization, methodology, and writing and editing of the manuscript.

## Funding

VtR Inc-CGU (SCRPD1L0221); DOXABIO-CGU (SCRPD1K0131), and CGU grant (UZRPD1L0011, UZRPD1M0081).

## Declaration of competing interest

The author declares no conflict of interest, financial or otherwise.

## Data Availability

Data will be made available on request.

## References

[1] Cockwell P., Fisher L.A. (2020). The global burden of chronic kidney disease. The Lancet.

[2] Kovesdy C.P. (2022). Epidemiology of chronic kidney disease: an update 2022. Kidney Int. Suppl..

[3] Nowak N., Yamanouchi M., Satake E. (2022). The nephroprotective properties of extracellular vesicles in experimental models of chronic kidney disease: a systematic review. Stem Cell Rev Rep.

[4] Hill N.R., Fatoba S.T., Oke J.L., Hirst J.A., O'Callaghan C.A., Lasserson D.S., Hobbs F.D.R. (2016). Global prevalence of chronic kidney disease – a systematic review and meta-analysis. PLoS One.

[5] Li J., Ma L., Yu H., Yao Y., Xu Z., Lin W., Wang L., Wang X., Yang H. (2022). MicroRNAs as potential biomarkers for the diagnosis of chronic kidney disease: a systematic review and meta-analysis. Front. Med..

[6] Breyer M.D., Susztak K. (2016). Developing treatments for chronic kidney disease in the 21st century. Semin. Nephrol..

[7] van der Pol E., Böing A.N., Gool E.L., Nieuwland R. (2016). Recent developments in the nomenclature, presence, isolation, detection and clinical impact of extracellular vesicles. J. Thromb. Haemostasis.

[8] Ix, J., Shlipak, M.: Trial of Pirfenidone to Prevent Progression of Chronic Kidney Disease (TOP?CKD).

[9] Foreman K.J., Marquez N., Dolgert A., Fukutaki K., Fullman N., McGaughey M., Pletcher M.A., Smith A.E., Tang K., Yuan C.W., Brown J.C., Friedman J., He J., Heuton K.R., Holmberg M., Patel D.J., Reidy P., Carter A., Cercy K., Chapin A., Douwes-Schultz D., Frank T., Goettsch F., Liu P.Y., Nandakumar V., Reitsma M.B., Reuter V., Sadat N., Sorensen R.J.D., Srinivasan V., Updike R.L., York H., Lopez A.D., Lozano R., Lim S.S., Mokdad A.H., Vollset S.E., Murray C.J.L. (2018). Forecasting life expectancy, years of life lost, and all-cause and cause-specific mortality for 250 causes of death: reference and alternative scenarios for 2016–40 for 195 countries and territories. The Lancet.

[10] Ruiz-Ortega M., Rayego-Mateos S., Lamas S., Ortiz A., Rodrigues-Diez R.R. (2020). Targeting the progression of chronic kidney disease. Nat. Rev. Nephrol..

[11] O'Brien K., Breyne K., Ughetto S., Laurent L.C., Breakefield X.O. (2020). RNA delivery by extracellular vesicles in mammalian cells and its applications. Nat. Rev. Mol. Cell Biol..

[12] Sundström J., Bodegard J., Bollmann A., Vervloet M.G., Mark P.B., Karasik A., Taveira-Gomes T., Botana M., Birkeland K.I., Thuresson M., Jäger L., Sood M.M., VanPottelbergh G., Tangri N. (2022). Prevalence, outcomes, and cost of chronic kidney disease in a contemporary population of 2·4 million patients from 11 countries: the CaReMe CKD study. The Lancet Regional Health - Europe.

[13] New global kidney health report sheds light on current capacity around the world to deliver kidney care.

[14] Wu M.Y., Wu M.S. (2018). Taiwan renal care system: a learning health-care system. Nephrology (Carlton).

[15] Wen C.P., Cheng T.Y.D., Tsai M.K., Chang Y.C., Chan H.T., Tsai S.P., Chiang P.H., Hsu C.C., Sung P.K., Hsu Y.H., Wen S.F. (2008). All-cause mortality attributable to chronic kidney disease: a prospective cohort study based on 462 293 adults in Taiwan. The Lancet.

[16] Bikbov B., Purcell C.A., Levey A.S., Smith M., Abdoli A., Abebe M., Adebayo O.M., Afarideh M., Agarwal S.K., Agudelo-Botero M., Ahmadian E., Al-Aly Z., Alipour V., Almasi-Hashiani A., Al-Raddadi R.M., Alvis-Guzman N., Amini S., Andrei T., Andrei C.L., Andualem Z., Anjomshoa M., Arabloo J., Ashagre A.F., Asmelash D., Ataro Z., Atout M.M.d.W., Ayanore M.A., Badawi A., Bakhtiari A., Ballew S.H., Balouchi A., Banach M., Barquera S., Basu S., Bayih M.T., Bedi N., Bello A.K., Bensenor I.M., Bijani A., Boloor A., Borzì A.M., Cámera L.A., Carrero J.J., Carvalho F., Castro F., Catalá-López F., Chang A.R., Chin K.L., Chung S.C., Cirillo M., Cousin E., Dandona L., Dandona R., Daryani A., Das Gupta R., Demeke F.M., Demoz G.T., Desta D.M., Do H.P., Duncan B.B., Eftekhari A., Esteghamati A., Fatima S.S., Fernandes J.C., Fernandes E., Fischer F., Freitas M., Gad M.M., Gebremeskel G.G., Gebresillassie B.M., Geta B., Ghafourifard M., Ghajar A., Ghith N., Gill P.S., Ginawi I.A., Gupta R., Hafezi-Nejad N., Haj-Mirzaian A., Haj-Mirzaian A., Hariyani N., Hasan M., Hasankhani M., Hasanzadeh A., Hassen H.Y., Hay S.I., Heidari B., Herteliu C., Hoang C.L., Hosseini M., Hostiuc M., Irvani S.S.N., Islam S.M.S., Jafari Balalami N., James S.L., Jassal S.K., Jha V., Jonas J.B., Joukar F., Jozwiak J.J., Kabir A., Kahsay A., Kasaeian A., Kassa T.D., Kassaye H.G., Khader Y.S., Khalilov R., Khan E.A., Khan M.S., Khang Y.H., Kisa A., Kovesdy C.P., Kuate Defo B., Kumar G.A., Larsson A.O., Lim L.L., Lopez A.D., Lotufo P.A., Majeed A., Malekzadeh R., März W., Masaka A., Meheretu H.A.A., Miazgowski T., Mirica A., Mirrakhimov E.M., Mithra P., Moazen B., Mohammad D.K., Mohammadpourhodki R., Mohammed S., Mokdad A.H., Morales L., Moreno Velasquez I., Mousavi S.M., Mukhopadhyay S., Nachega J.B., Nadkarni G.N., Nansseu J.R., Natarajan G., Nazari J., Neal B., Negoi R.I., Nguyen C.T., Nikbakhsh R., Noubiap J.J., Nowak C., Olagunju A.T., Ortiz A., Owolabi M.O., Palladino R., Pathak M., Poustchi H., Prakash S., Prasad N., Rafiei A., Raju S.B., Ramezanzadeh K., Rawaf S., Rawaf D.L., Rawal L., Reiner R.C., Rezapour A., Ribeiro D.C., Roever L., Rothenbacher D., Rwegerera G.M., Saadatagah S., Safari S., Sahle B.W., Salem H., Sanabria J., Santos I.S., Sarveazad A., Sawhney M., Schaeffner E., Schmidt M.I., Schutte A.E., Sepanlou S.G., Shaikh M.A., Sharafi Z., Sharif M., Sharifi A., Silva D.A.S., Singh J.A., Singh N.P., Sisay M.M.M., Soheili A., Sutradhar I., Teklehaimanot B.F., Tesfay B. etsay, Teshome G.F., Thakur J.S., Tonelli M., Tran K.B., Tran B.X., Tran Ngoc C., Ullah I., Valdez P.R., Varughese S., Vos T., Vu L.G., Waheed Y., Werdecker A., Wolde H.F., Wondmieneh A.B., Wulf Hanson S., Yamada T., Yeshaw Y., Yonemoto N., Yusefzadeh H., Zaidi Z., Zaki L., Zaman S. Bin, Zamora N., Zarghi A., Zewdie K.A., Ärnlöv J., Coresh J., Perico N., Remuzzi G., Murray C.J.L. (2020). Global, regional, and national burden of chronic kidney disease, 1990–2017: a systematic analysis for the Global Burden of Disease Study 2017. The Lancet.

[17] Lai T.S., Hsu C.C., Lin M.H., Wu V.C., Chen Y.M. (2022). Trends in the incidence and prevalence of end-stage kidney disease requiring dialysis in Taiwan: 2010–2018. J. Formos. Med. Assoc..

[18] Wang H.S., Yi M.Y., Wu X., Liu Q., Deng Y.H., Wu T., Wang L., Kang Y.X., Luo X.Q., Yan P., Wang M., Duan S. Bin (2022). Effects of mesenchymal stem cells in renovascular disease of preclinical and clinical studies: a systematic review and meta-analysis. Sci. Rep..

[19] Zhou T., Liao C., Li H.Y., Lin W., Lin S., Zhong H. (2020). Efficacy of mesenchymal stem cells in animal models of lupus nephritis: a meta-analysis. Stem Cell Res. Ther..

[20] Xia Y., Ye H., Li K., Shi B., Sun X., Wu J. (2023). Efficacy of mesenchymal stem cell therapy on lupus nephritis and renal function in systemic lupus Erythematosus: a meta-analysis. Clin. Invest. Med..

[21] Chavda V.P., Luo G., Bezbaruah R., Kalita T., Sarma A., Deka G., Duo Y., Das B.K., Shah Y., Postwala H. (2024). Unveiling the promise: exosomes as game-changers in anti-infective therapy. Explorations.

[22] Zhao S., Li W., Yu W., Rao T., Li H., Ruan Y., Yuan R., Li C., Ning J., Li S., Chen W., Cheng F., Zhou X. (2021). Exosomal miR-21 from tubular cells contributes to renal fibrosis by activating fibroblasts via targeting PTEN in obstructed kidneys. Theranostics.

[23] Liu Y., Guo Y., Bao S., Huang H., Liu W., Guo W. (2022). Bone marrow mesenchymal stem cell-derived exosomal microRNA-381-3p alleviates vascular calcification in chronic kidney disease by targeting NFAT5. Cell Death Dis..

[24] Wang Y., Guo Y.F., Fu G.P., Guan C., Zhang X., Yang D.G., Shi Y.C. (2020). Protective effect of miRNA-containing extracellular vesicles derived from mesenchymal stromal cells of old rats on renal function in chronic kidney disease. Stem Cell Res. Ther..

[25] Li D., Qu J., Yuan X., Zhuang S., Wu H., Chen R., Wu J., Zhang M., Ying L. (2022). Mesenchymal stem cells alleviate renal fibrosis and inhibit Autophagy via exosome transfer of miRNA-122a. Stem Cell. Int..

[26] Pomatto M.A.C., Gai C., Bussolati B., Camussi G. (2017). Extracellular vesicles in renal pathophysiology. Front. Mol. Biosci..

[27] Karpman D., Ståhl A.L., Arvidsson I. (2017). Extracellular vesicles in renal disease. Nat. Rev. Nephrol..

[28] Lv L.-L., Feng Y., Tang T.-T., Liu B.-C., Lin-Li Lv C., Hospital Z. (2019). New insight into the role of extracellular vesicles in kidney disease. J. Cell Mol. Med..

[29] Kim D.K., Lee J., Kim S.R., Choi D.S., Yoon Y.J., Kim J.H., Go G., Nhung D., Hong K., Jang S.C., Kim S.H., Park K.S., Kim O.Y., Park H.T., Seo J.H., Aikawa E., Baj-Krzyworzeka M., Van Balkom B.W.M., Belting M., Blanc L., Bond V., Bongiovanni A., Borràs F.E., Buée L., Buzás E.I., Cheng L., Clayton A., Cocucci E., Dela Cruz C.S., Desiderio D.M., Di Vizio D., Ekström K., Falcon-Perez J.M., Gardiner C., Giebel B., Greening D.W., Christina Gross J., Gupta D., Hendrix A., Hill A.F., Hill M.M., Nolte-’T Hoen E., Hwang D.W., Inal J., Jagannadham M.V., Jayachandran M., Jee Y.K., Jørgensen M., Kim K.P., Kim Y.K., Kislinger T., Lässer C., Lee D.S., Lee H., Van Leeuwen J., Lener T., Liu M.L., Lötvall J., Marcilla A., Mathivanan S., Möller A., Morhayim J., Mullier F., Nazarenko I., Nieuwland R., Nunes D.N., Pang K., Park J., Patel T., Pocsfalvi G., Del Portillo H., Putz U., Ramirez M.I., Rodrigues M.L., Roh T.Y., Royo F., Sahoo S., Schiffelers R., Sharma S., Siljander P., Simpson R.J., Soekmadji C., Stahl P., Stensballe A., Stepień E., Tahara H., Trummer A., Valadi H., Vella L.J., Wai S.N., Witwer K., Yánez-Mó M., Youn H., Zeidler R., Gho Y.S. (2015). EVpedia: a community web portal for extracellular vesicles research. Bioinformatics.

[30] O'Brien J., Hayder H., Zayed Y., Peng C. (2018). Overview of MicroRNA Biogenesis, mechanisms of actions, and Circulation. Front. Endocrinol..

[31] Panda B., Sharma Y., Gupta S., Mohanty S. (2021). Mesenchymal stem cell-derived exosomes as an emerging Paradigm for regenerative therapy and Nano-medicine: a comprehensive review. Life.

[32] Reinders M.E.J., de Fijter J.W., Roelofs H., Bajema I.M., de Vries D.K., Schaapherder A.F., Claas F.H.J., van Miert P.P.M.C., Roelen D.L., van Kooten C., Fibbe W.E., Rabelink T.J. (2013). Autologous bone marrow-derived mesenchymal stromal cells for the treatment of Allograft Rejection after renal Transplantation: results of a phase I study. Stem Cells Transl. Med..

[33] Nowak N., Yamanouchi M., Satake E. (2022). The Nephroprotective properties of extracellular vesicles in experimental models of chronic kidney disease: a systematic review. Stem Cell Rev Rep.

[34] Lu Y., Zhang R., Gu X., Wang X., Xi P., Chen X. (2023). Exosomes from tubular epithelial cells undergoing epithelial-to-mesenchymal transition promote renal fibrosis by M1 macrophage activation. FASEB Bioadv.

[35] Wang Z., Yu Y., Jin L., Tan X., Liu B., Zhang Z., Wang Z., Long C., Shen L., Wei G., He D. (2023). HucMSC exosomes attenuate partial bladder outlet obstruction-induced renal injury and cell proliferation via the Wnt/β-catenin pathway. Eur. J. Pharmacol..

[36] Liu Y., Guo W., Guo Y., Chen X., Liu W. (2022). Bone marrow mesenchymal stem cell-derived exosomes improve renal fibrosis via regulating Smurf 2/Smad 7. Frontiers in Bioscience - Landmark.

[37] Alasmari W.A., Abdelfattah-Hassan A., El-Ghazali H.M., Abdo S.A., Ibrahim D., Elsawy N.A., El-Shetry E.S., Saleh A.A., Abourehab M.A.S., Mahfouz H. (2022). Exosomes derived from BM-MSCs mitigate the development of chronic kidney damage post-Menopause via Interfering with fibrosis and apoptosis. Biomolecules.

[38] Liang M., Zhang D., Zheng D., He W., Jin J. (2022). Exosomes from miR-374a-5p-modified mesenchymal stem cells inhibit the progression of renal fibrosis by regulating MAPK6/MK5/YAP axis. Bioengineered.

[39] Zhao M., Yang B., Li L., Si Y., Chang M., Ma S., Li R., Wang Y., Zhang Y. (2022). Efficacy of Modified Huangqi Chifeng decoction in alleviating renal fibrosis in rats with IgA nephropathy by inhibiting the TGF-β1/Smad3 signaling pathway through exosome regulation. J. Ethnopharmacol..

[40] Zheng H., Ji J., Zhao T., Wang E., Zhang A. (2023). Exosome-encapsulated miR-26a attenuates aldosterone-induced tubulointerstitial fibrosis by inhibiting the CTGF/SMAD3 signaling pathway. Int. J. Mol. Med..

[41] Yea J.H., Yoon Y.M., Lee J.H., Yun C.W., Lee S.H. (2021). Exosomes isolated from melatonin-stimulated mesenchymal stem cells improve kidney function by regulating inflammation and fibrosis in a chronic kidney disease mouse model. J. Tissue Eng..

[42] Yin D., Cao J.Y., Yang Y., Li Z.T., Liu H., Tang T.T., Ni W.J., Zhang Y.L., Jiang W., Wen Y., Li Z.L., Zhao J., Lv L.L., Liu B.C., Wang B. (2023). Quercetin alleviates tubulointerstitial inflammation by inhibiting exosomes-mediated crosstalk between tubular epithelial cells and macrophages. Inflamm. Res..

[43] Liu X., Liu Z., Wang C., Miao J., Zhou S., Ren Q., Jia N., Zhou L., Liu Y. (2023). Kidney tubular epithelial cells control interstitial fibroblast fate by releasing TNFAIP8-encapsulated exosomes. Cell Death Dis..

[44] (2020). Exosomes Released by Human Umbilical Cord Mesenchymal Stem Cells Protect against Renal Interstitial Fibrosis through ROS Mediated P38MAPK ERK Signaling Pathway.

[45] Liu X., Miao J., Wang C., Zhou S., Chen S., Ren Q., Hong X., Wang Y., Hou F.F., Zhou L., Liu Y. (2020). Tubule-derived exosomes play a central role in fibroblast activation and kidney fibrosis. Kidney Int..

[46] Wan Y., Yu Y., Yu C., Luo J., Wen S., Shen L., Wei G., Hua Y. (2023). Human umbilical cord mesenchymal stem cell exosomes alleviate acute kidney injury by inhibiting pyroptosis in rats and NRK-52E cells. Ren. Fail..

[47] Jin J., Qian F., Zheng D., He W., Gong J., He Q. (2021). Mesenchymal stem cells attenuate renal fibrosis via exosomes-mediated delivery of microrna let-7i-5p antagomir. Int. J. Nanomed..

[48] Liu L., Wu Y., Wang P., Shi M., Wang J., Ma H., Sun D. (2021). PSC-MSC-Derived exosomes Protect against kidney fibrosis in Vivo and in vitro through the SIRT6/β-catenin signaling pathway. Int J Stem Cells.

[49] Wang H., Wang B., Zhang A., Hassounah F., Seow Y., Wood M., Ma F., Klein J.D., Price S.R., Wang X.H. (2019). Exosome-mediated miR-29 transfer reduces muscle atrophy and kidney fibrosis in mice. Mol. Ther..

[50] PRISMA, http://www.prisma-statement.org/?AspxAutoDetectCookieSupport=1.

[51] Jiang H., Li J., Yu K., Yang H., Min X., Chen H., Wu T. (2017). Associations of estimated glomerular filtration rate and blood urea nitrogen with incident coronary heart disease: the Dongfeng-Tongji Cohort Study. Sci. Rep..

[52] Du S., Guan Y., Xie A., Yan Z., Gao S., Li W., Rao L., Chen X., Chen T. (2023). Extracellular vesicles: a rising star for therapeutics and drug delivery. J. Nanobiotechnol..

[53] Home | ARRIVE Guidelines, https://arriveguidelines.org/.

[54] Théry C., Witwer K.W., Aikawa E., Alcaraz M.J., Anderson J.D., Andriantsitohaina R., Antoniou A., Arab T., Archer F., Atkin-Smith G.K., Ayre D.C., Bach J.M., Bachurski D., Baharvand H., Balaj L., Baldacchino S., Bauer N.N., Baxter A.A., Bebawy M., Beckham C., Bedina Zavec A., Benmoussa A., Berardi A.C., Bergese P., Bielska E., Blenkiron C., Bobis-Wozowicz S., Boilard E., Boireau W., Bongiovanni A., Borràs F.E., Bosch S., Boulanger C.M., Breakefield X., Breglio A.M., Brennan M., Brigstock D.R., Brisson A., Broekman M.L.D., Bromberg J.F., Bryl-Górecka P., Buch S., Buck A.H., Burger D., Busatto S., Buschmann D., Bussolati B., Buzás E.I., Byrd J.B., Camussi G., Carter D.R.F., Caruso S., Chamley L.W., Chang Y.T., Chaudhuri A.D., Chen C., Chen S., Cheng L., Chin A.R., Clayton A., Clerici S.P., Cocks A., Cocucci E., Coffey R.J., Cordeiro-da-Silva A., Couch Y., Coumans F.A.W., Coyle B., Crescitelli R., Criado M.F., D'Souza-Schorey C., Das S., de Candia P., De Santana E.F., De Wever O., del Portillo H.A., Demaret T., Deville S., Devitt A., Dhondt B., Di Vizio D., Dieterich L.C., Dolo V., Dominguez Rubio A.P., Dominici M., Dourado M.R., Driedonks T.A.P., Duarte F.V., Duncan H.M., Eichenberger R.M., Ekström K., El Andaloussi S., Elie-Caille C., Erdbrügger U., Falcón-Pérez J.M., Fatima F., Fish J.E., Flores-Bellver M., Försönits A., Frelet-Barrand A., Fricke F., Fuhrmann G., Gabrielsson S., Gámez-Valero A., Gardiner C., Gärtner K., Gaudin R., Gho Y.S., Giebel B., Gilbert C., Gimona M., Giusti I., Goberdhan D.C.I., Görgens A., Gorski S.M., Greening D.W., Gross J.C., Gualerzi A., Gupta G.N., Gustafson D., Handberg A., Haraszti R.A., Harrison P., Hegyesi H., Hendrix A., Hill A.F., Hochberg F.H., Hoffmann K.F., Holder B., Holthofer H., Hosseinkhani B., Hu G., Huang Y., Huber V., Hunt S., Ibrahim A.G.E., Ikezu T., Inal J.M., Isin M., Ivanova A., Jackson H.K., Jacobsen S., Jay S.M., Jayachandran M., Jenster G., Jiang L., Johnson S.M., Jones J.C., Jong A., Jovanovic-Talisman T., Jung S., Kalluri R., Kano S. ichi, Kaur S., Kawamura Y., Keller E.T., Khamari D., Khomyakova E., Khvorova A., Kierulf P., Kim K.P., Kislinger T., Klingeborn M., Klinke D.J., Kornek M., Kosanović M.M., Kovács Á.F., Krämer-Albers E.M., Krasemann S., Krause M., Kurochkin I.V., Kusuma G.D., Kuypers S., Laitinen S., Langevin S.M., Languino L.R., Lannigan J., Lässer C., Laurent L.C., Lavieu G., Lázaro-Ibáñez E., Le Lay S., Lee M.S., Lee Y.X.F., Lemos D.S., Lenassi M., Leszczynska A., Li I.T.S., Liao K., Libregts S.F., Ligeti E., Lim R., Lim S.K., Linē A., Linnemannstöns K., Llorente A., Lombard C.A., Lorenowicz M.J., Lörincz Á.M., Lötvall J., Lovett J., Lowry M.C., Loyer X., Lu Q., Lukomska B., Lunavat T.R., Maas S.L.N., Malhi H., Marcilla A., Mariani J., Mariscal J., Martens-Uzunova E.S., Martin-Jaular L., Martinez M.C., Martins V.R., Mathieu M., Mathivanan S., Maugeri M., McGinnis L.K., McVey M.J., Meckes D.G., Meehan K.L., Mertens I., Minciacchi V.R., Möller A., Møller Jørgensen M., Morales-Kastresana A., Morhayim J., Mullier F., Muraca M., Musante L., Mussack V., Muth D.C., Myburgh K.H., Najrana T., Nawaz M., Nazarenko I., Nejsum P., Neri C., Neri T., Nieuwland R., Nimrichter L., Nolan J.P., Nolte-’t Hoen E.N.M., Noren Hooten N., O'Driscoll L., O'Grady T., O'Loghlen A., Ochiya T., Olivier M., Ortiz A., Ortiz L.A., Osteikoetxea X., Ostegaard O., Ostrowski M., Park J., Pegtel D.M., Peinado H., Perut F., Pfaffl M.W., Phinney D.G., Pieters B.C.H., Pink R.C., Pisetsky D.S., Pogge von Strandmann E., Polakovicova I., Poon I.K.H., Powell B.H., Prada I., Pulliam L., Quesenberry P., Radeghieri A., Raffai R.L., Raimondo S., Rak J., Ramirez M.I., Raposo G., Rayyan M.S., Regev-Rudzki N., Ricklefs F.L., Robbins P.D., Roberts D.D., Rodrigues S.C., Rohde E., Rome S., Rouschop K.M.A., Rughetti A., Russell A.E., Saá P., Sahoo S., Salas-Huenuleo E., Sánchez C., Saugstad J.A., Saul M.J., Schiffelers R.M., Schneider R., Schøyen T.H., Scott A., Shahaj E., Sharma S., Shatnyeva O., Shekari F., Shelke G.V., Shetty A.K., Shiba K., Siljander P.R.M., Silva A.M., Skowronek A., Snyder O.L., Soares R.P., Sódar B.W., Soekmadji C., Sotillo J., Stahl P.D., Stoorvogel W., Stott S.L., Strasser E.F., Swift S., Tahara H., Tewari M., Timms K., Tiwari S., Tixeira R., Tkach M., Toh W.S., Tomasini R., Torrecilhas A.C., Tosar J.P., Toxavidis V., Urbanelli L., Vader P., van Balkom B.W.M., van der Grein S.G., Van Deun J., van Herwijnen M.J.C., Van Keuren-Jensen K., van Niel G., van Royen M.E., van Wijnen A.J., Vasconcelos M.H., Vechetti I.J., Veit T.D., Vella L.J., Velot É., Verweij F.J., Vestad B., Viñas J.L., Visnovitz T., Vukman K.V., Wahlgren J., Watson D.C., Wauben M.H.M., Weaver A., Webber J.P., Weber V., Wehman A.M., Weiss D.J., Welsh J.A., Wendt S., Wheelock A.M., Wiener Z., Witte L., Wolfram J., Xagorari A., Xander P., Xu J., Yan X., Yáñez-Mó M., Yin H., Yuana Y., Zappulli V., Zarubova J., Žėkas V., Zhang J. ye, Zhao Z., Zheng L., Zheutlin A.R., Zickler A.M., Zimmermann P., Zivkovic A.M., Zocco D., Zuba-Surma E.K. (2018). Minimal information for studies of extracellular vesicles 2018 (MISEV2018): a position statement of the International Society for Extracellular Vesicles and update of the MISEV2014 guidelines. J. Extracell. Vesicles.

[55] Baek G., Choi H., Kim Y., Lee H.C., Choi C. (2019). Mesenchymal stem cell-derived extracellular vesicles as therapeutics and as a drug delivery platform. Stem Cells Transl. Med..

