# Supplementary material for: Meta-analysis study of the therapeutic impact of Mesenchymal stem cells derived exosomes for chronic kidney diseases
Source: Biochem Biophys Rep. 2025 Jun 3;43:102072. doi: 10.1016/j.bbrep.2025.102072 (PMC12169747; doi:10.1016/j.bbrep.2025.102072)
Supplement: Multimedia component 2 [file mmc2.docx]

1. **BUN level**


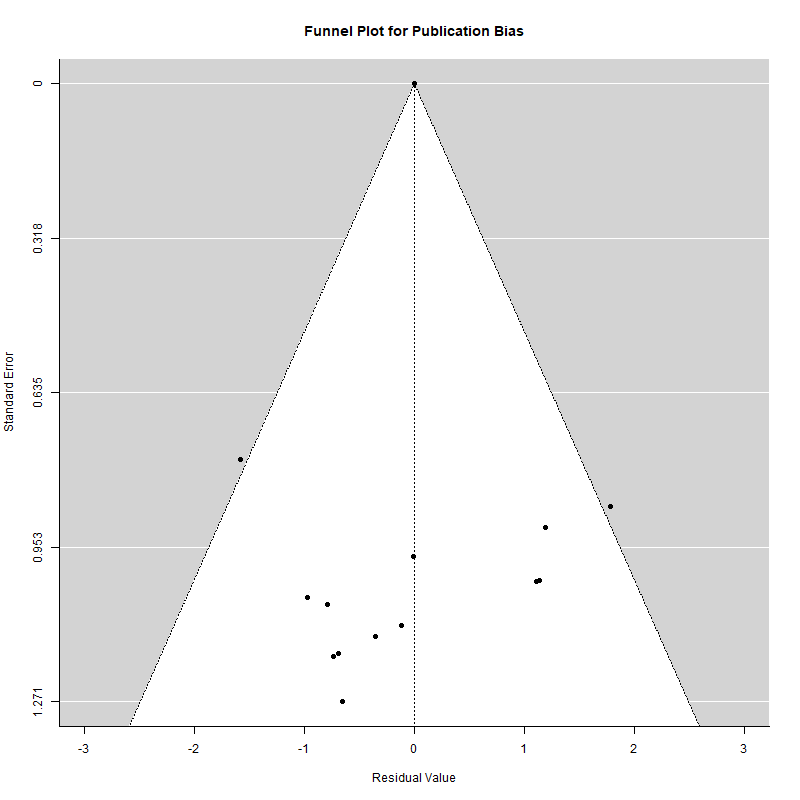


1. **SCR level**


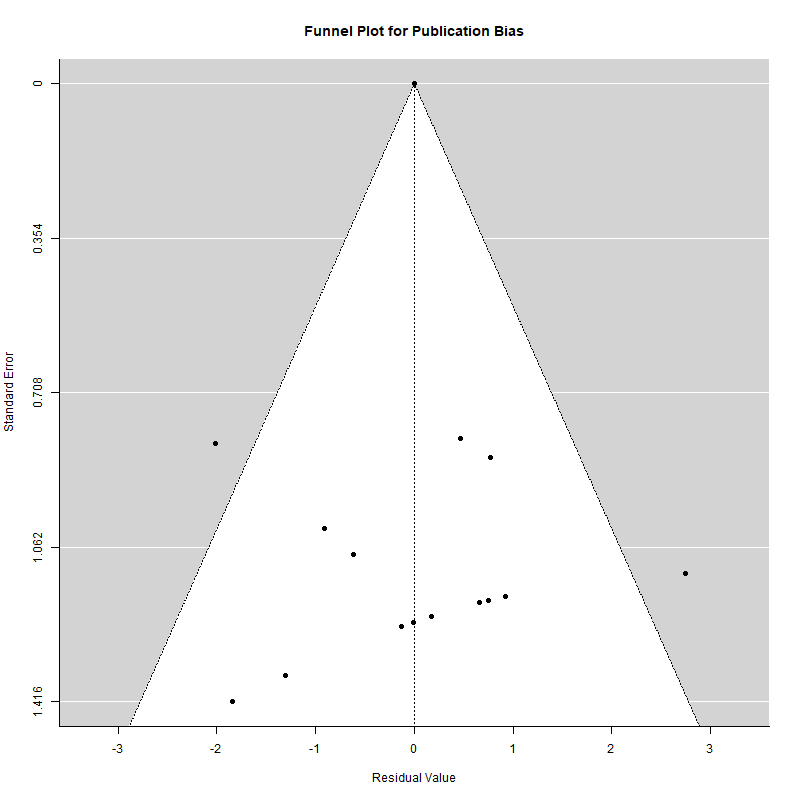


**Supplementary figure 3:** Funnel plot analysis based on animal model with their 95% confidence intervals (A) efficacy in BUN reduction of MSC derived EXOs (B) efficacy in SCR reduction of MSC derived EXOs

1. **BUN level**


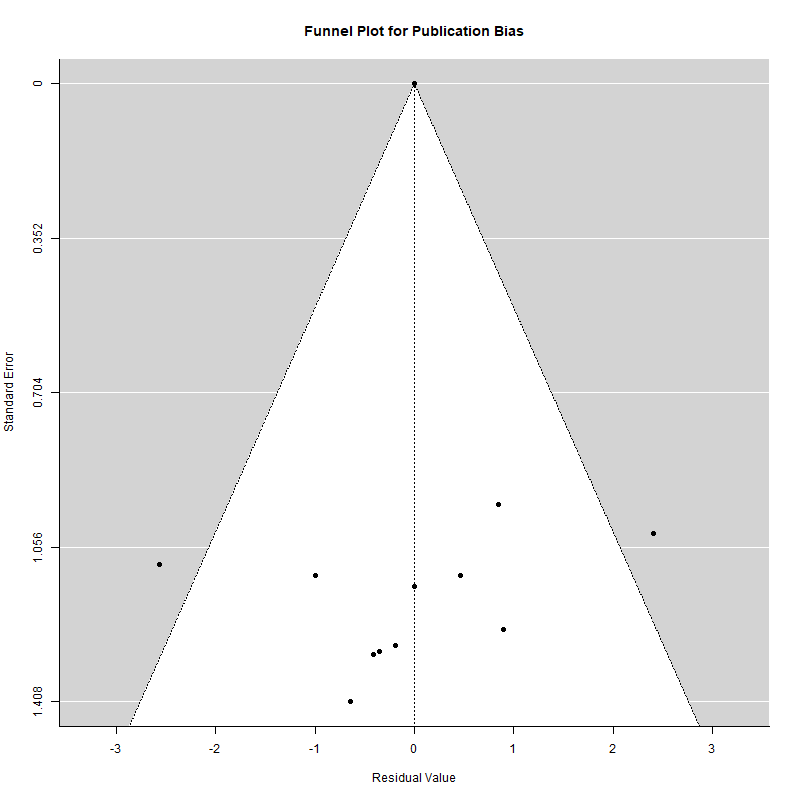


1. **SCR level**


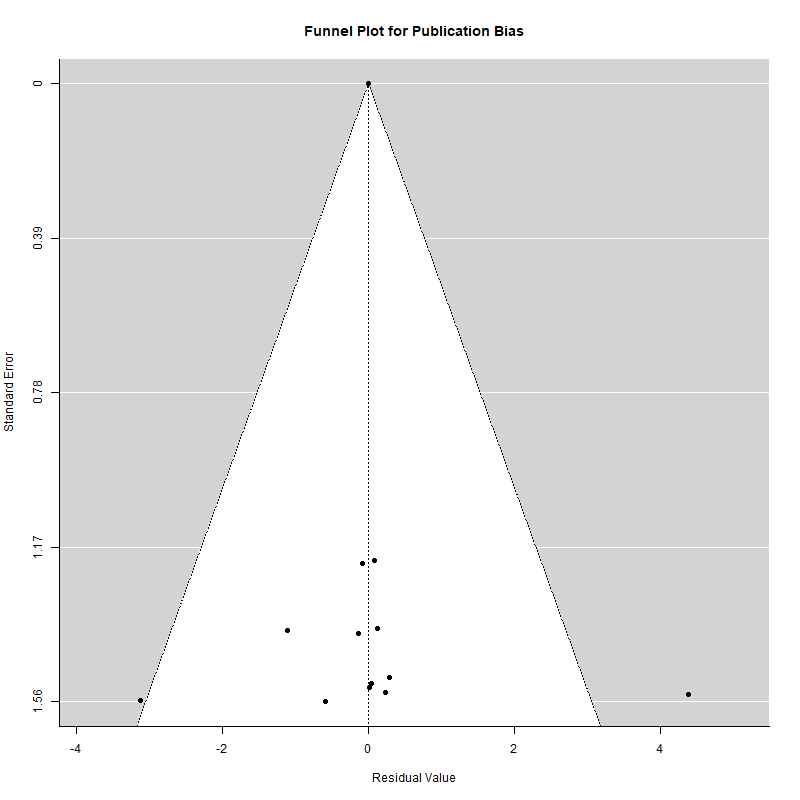


**Supplementary figure 4:** Funnel plot analysis based on exosome source with their 95% confidence intervals (A) efficacy in BUN reduction of MSC derived EXOs (B) efficacy in SCR reduction of MSC derived EXOs
